# Leukocytes in Inflammation, Resolution of Inflammation, Autoimmune Diseases and Cancer

**DOI:** 10.3390/cells10071735

**Published:** 2021-07-09

**Authors:** János G. Filep

**Affiliations:** Department of Pathology and Cell Biology, University of Montreal, Montreal, QC H1T 2M4, Canada; janos.g.filep@umontreal.ca

Inflammation is a double-edged sword. Acute inflammation is broadly defined as a protective response of the organism to invading pathogens and tissue injury. The reaction should ideally be localized and self-limited, leading to the elimination of pathogens and damaged cells, and the clearance of inflammatory cells, followed by tissue repair and return to homeostasis [1,2]. Excessive inflammation or failure of the initial response to resolve in a timely manner results in nonresolving inflammation, which is now considered the critical component of many chronic human diseases, including atherosclerosis, arthritis, pulmonary diseases, autoimmunity, diabetes and cancer [3]. The inflammatory response is a complex process, heterogeneous in nature and depends on the type of disease and organ in which it occurs. The sequence of events and signaling circuits initiated during inflammation and the dual nature of inflammation has received considerable attention and is summarized in guiding maps for inflammation [4] and resolution pathways [5]. However, accumulating data expand and challenge many of our current concepts. Thus, identifying macrophage and neutrophil functional heterogeneity, the characterization of novel mechanisms and the recognition of resolution as an active process governed by specialized pro-resolving mediators shed new light on the roles of leukocyte subsets in acute and chronic inflammatory conditions. Since the inflammatory response is critical for survival, the current anti-inflammatory therapies have limitations and often do not lead to the repair of affected tissues. A better understanding of inflammatory and resolution pathways will likely enable further therapeutic advances. This Special Issue of *Cells* covers a broad spectrum of diseases, including rheumatoid arthritis-associated cardiovascular diseases, chronic respiratory diseases, acute-on-chronic liver failure, pregnancy-associated hypertension and eclampsia, and autoimmune diseases, in which leukocytes play critical pathophysiological roles. This collection of articles, both review and original research papers, highlights recent advances in elucidating leukocyte-centered mechanisms under these pathological conditions and discuss new avenues for potential therapeutic interventions to promote repair and return to homeostasis.

Rheumatoid arthritis is an autoimmune disease characterized by inflammation within synovial joints and an independent risk factor for cardiovascular diseases. Chen and colleagues [6] review the link between inflammation and non-ischemic cardiac diseases in patients with rheumatoid arthritis, with a particular focus on heart failure with preserved ejection fraction (HFpEF). HFpEF likely represents a distinct mechanism of heart failure, as therapies for heart failure with reduced ejection fraction have limited success in HFpEF patients. The authors argue that local or systemic inflammation may either directly affect the heart or render the heart more susceptible to traditional risk factors. Indeed, rheumatoid arthritis is associated with increased circulating levels of pro-inflammatory mediators, which promote the recruitment of leukocytes into cardiac tissue, in particular MHCII^high^ macrophages, evoke oxidative stress, cardiac muscle hypertrophy and increased stiffness, ultimately leading to diastolic dysfunction. While a number of biological and synthetic disease modifying antirheumatic drugs, non-steroidal anti-inflammatory drugs and corticosteroids can improve clinical symptoms, they have little impact on the development of heart failure, or some drugs could even increase the risk of non-ischemic heart failure. As the progression of rheumatoid arthritis is now increasingly recognized as a result of defective resolution mechanism [7], failed resolution pathways could also underlie the development of HFpEF. The authors analyze preclinical data, indicating the cardioprotective effects of formyl-peptide receptor 2 (FPR2) agonists lipoxin A_4_, resolvin D1, annexin A1 and the synthetic compound 17b (CMPD17b). Another option is the use of statins, which are generally prescribed to lower plasma cholesterol level. However, statins have been shown to increase the production of 15-epi-LXA_4_ and 13-series resolvins, thereby correcting the inflammation resolution deficit independent of their lipid-lowering capacity.

Casulleras and colleagues [8] describe the prevailing characteristics of the hyperinflammatory state underlying the development of acute-on-chronic liver failure in patients, which is associated with high short-term mortality. Although, at present, only limited information is available on the triggers of acute-on-chronic liver failure, inappropriate response of both the innate and adaptive immune system (leading to altered function and metabolism in neutrophils, monocytes, METK^+^ macrophages and NK cells) to pathogen-associated molecular patterns and/or damage-associated molecular patterns drive the rapid deterioration in liver function. This response is frequently associated with an immunosuppression-like state in patients, possibly due to the presence of a more tolerogenic phenotype of immune cells in the liver, which is not present in circulating counterparts. In addition to liver transplantation, the most efficient therapy for acute-on-chronic liver failure, the authors also discuss several potential therapeutic interventions to limit the deleterious consequences of misbalanced immune reactions. Among these options are treatment with human serum albumin that exerts immunomodulatory actions in leukocytes by blocking Toll-like receptor signaling pathways in the endosomal compartment [9], TLR4 antagonists, IL-22, G-CSF and stem cell therapy.

The lung has long been recognized to have a significant reparative capacity in response to injury. Dysregulated inflammation and aberrant or defective repair mechanisms are increasingly being recognized as central events in several chronic pulmonary pathologies, including chronic obstructive pulmonary disease (COPD), asthma, pulmonary fibrosis and acute respiratory distress syndrome (ARDS) [10]. Croasdell Lucchini and colleagues [11] review the plasticity of respiratory epithelial cells to respond to injury and to activate injury-specific regenerative processes through crosstalk with immune cells (particularly granulocytes and macrophages). Epithelial crosstalk with other cell types is mediated by altered cytokine profiles, the secretion of growth factors, and the generation of pro-resolving mediators, extracellular vesicles and miRNAs. Preclinical models indicate the beneficial actions of the therapeutic targeting of soluble mediators, signaling cascades and epithelial repair mechanisms; however, translating these approaches to the clinical setting will require further studies. 

Accumulating data indicate a role for inflammation in the development of pregnancy-induced hypertension and preeclampsia. Socha et al. [12] discuss how the activation of the NLRP3 inflammasome by hypercholesterolemia, hyperglycemia or hyperuricemia initiates a feed-forward loop, consisting of local inflammation, sympathetic outflow and increased production of angiotensin II, which leads to hypertension and vascular injury. NLRP3 activation also underpins renal injury, enhanced coagulation and thrombus formation, and placental abruption, hallmarks of preeclampsia. These findings identify the NLRP3 inflammasome as a potential therapeutic target for the prevention and/or treatment of pregnancy-induced hypertension and preeclampsia. 

Sanches and colleagues [13] report a dual role for the glucocorticoid-regulated protein annexin A1 in the regulation of the NLRP3 inflammasome in murine neutrophils. Neutrophils are an important source of, and cellular targets for, annexin A1 [14]. The genetic deletion of annexin A1 resulted in reduced IL-1β release and altered the lipid profile, indicating a role for endogenous annexin A1 in the proper activation of NLRP3 machinery. By contrast, the treatment of neutrophils with annexin A1-derived peptide Ac2-26 markedly suppressed NLRP3 activation by nigericin or ATP parallel with increases in the levels of phosphatidylserine and oxidized phosphocholines, known biomarkers of the inflammation resolution circuits. Based on previous reports, the authors suggest that peptide Ac2-26 triggers the production of anti-inflammatory microvesicles and activates the intrinsic pro-apoptosis program in neutrophils. 

Similar to T cell and macrophage subsets, accumulating evidence indicates the unexpected heterogeneity and functional versatility of neutrophil granulocytes [15]. Changes in the neutrophil secretome, including cytokines, granular proteins, neutrophil extracellular traps and extracellular vesicles have received considerable attention, likely because of their importance in the inflammatory process. Kolonics and colleagues [16] provide an overview of extracellular vesicles as intercellular communication tools in homeostasis and under pathological conditions, including tumor progression, rheumatoid arthritis, autoimmunity and neurodegenerative diseases. The authors detail the composition and functional heterogeneity of neutrophil-derived extracellular vesicles, ranging from the spontaneous release of extracellular vesicles from unstimulated cells to those released in response to pathogens, bacterial constituents, pro-inflammatory mediators, pharmacological or environmental stimuli. In general, extracellular vesicles derived from resting and apoptotic neutrophils tend to send anti-inflammatory signals to neighboring cells, whereas activated neutrophils tend to release extracellular vesicles that generate pro-inflammatory signals to facilitate host defense. Since extracellular vesicles derived from the same neutrophil population can have diverse and sometimes even opposing actions on different target cells, depending on the cues received from the inflammatory microenvironment, the authors propose a continuous spectrum concept, where the released extracellular vesicles reflect the prevailing state of the parent cells. This concept may also raise the possibility of monitoring neutrophil extracellular vesicle composition as a potential biomarker and developing anti-inflammatory neutrophil extracellular vesicles as a therapeutic tool. 

Tumor cell-derived exosomes have long been recognized to mediate cellular crosstalk within the tumor microenvironment to drive tumor progression [17]. Making another step forward, Pritchard and colleagues [18] report that lung adenocarcinoma cell-derived exosomes induced transcriptional changes and reprogrammed metabolism in non-committed (M0) macrophages, resulting in their polarization to the M2 phenotype in vitro. Likewise, the co-culture of murine bone marrow cells and bone marrow-derived myeloid-derived suppressor cells (MDSCs) with Lewis lung carcinoma (LLC)-derived exosomes resulted in M2 macrophages. Interestingly, p53 regulated exosome production, whereas macrophage polarization occurred in a p53-independent fashion. These findings reveal a regulatory role of tumor-derived exosomes in promoting the polarization of tumor-associated macrophages in the lung towards the M2 phenotype, leading to immune suppression and the promotion of tumor growth.

Alternately activated M2-like macrophages also govern the remodeling of the extracellular matrix, a characteristic feature of many chronic respiratory diseases, such as idiopathic pulmonary fibrosis, chronic obstructive pulmonary diseases (COPD) and severe asthma [10]. Ho and colleagues [19] report that the transient pulmonary overexpression of oncostatin M or IL-6, members of the gp130 cytokine family, induced the expression of resistin-like molecule alpha (RELMα or FIZZ1, found in inflammatory zone 1), independent of IL-6 or STAT6 in mouse airway epithelial cells. These mechanisms contrast STAT6-mediated actions of oncostatin M on type 2 alveolar epithelial cells and the recruitment and polarization of macrophages towards the M2 phenotype, leading to RELMα release and the subsequent induction of extracellular matrix modulating genes. Although the human homolog for mouse RELMα has yet to be identified, these findings call for further studies on the role of RELM family proteins in lung pathologies associated with elevated oncostatin M levels.

As highlighted by these and numerous other previous studies, macrophage subsets are attractive therapeutic targets for the treatment of a wide range of common pathologies [20]. Poltavets and colleagues [21] provide an overview of key signaling molecules from surface markers through receptors and chemokines/cytokines to transcription factors, which characterize and drive M1 and M2 polarization. The authors argue that macrophages are easily reprogrammable cells and discuss strategies of target selection, methods of vector delivery and genome editing approaches to achieve the controllable promotion of the desired M1 or M2 phenotype, depending on the pathological conditions. Two rapidly developing approaches to obtain macrophages, the induced pluripotent stem cell technologies (iPSCs) and the use of engineered transcription activator-like effector nucleases (TALEN), hold promise for repair-facilitating interventions. Generating iPSCs from peripheral blood monocytes is less invasive than the use of skin fibroblasts. The feasibility of TALEN-promoted insertion codon-optimized cDNA for the generation of functionally corrected monocytes and macrophages has recently been shown to efficiently correct a defect in the *CSF2RA* gene, which results in life-threatening hereditary pulmonary alveolar proteinosis. 

Jin and colleagues [22] report that the infection of macrophages and lymphocytes to Theiler’s murine encephalomyelitis virus (TMEV), which induces an inflammatory demyelinating disease in susceptible mice similar to human multiple sclerosis, resulted in viral replication and the subsequent stimulation of dendritic cells, macrophages, B cells and T cells. TMEV-infected B cells exhibited elevated antigen-presenting function and produced autoantibodies. TMEV-infected antigen-presenting cells released excessive amounts of type I interferons, IL-6, IL-1 and prostaglandin E2, which contributed to a shift from the protective Th1 response to pathogenic Th17 response. These findings may imply a potentially important role for microbial infection in the induction or progression of autoimmune diseases.

Moerman-Herzog and colleagues [23] provide an overview of the clinical features and gene expression profiles aiming to distinguish benign and malignant lymphoproliferative phenotypes, in particular Sézary syndrome, an aggressive cutaneous T cell lymphoma, and lymphocytic-variant hypereosinophilic syndrome (L-HES). Shared features of these two syndromes include T cell proliferation and a Th2-like phenotype. *IL17RB* expression appears to be selective for L-HES, whereas Sézary syndrome is associated with the expression of a number of cancer-promoting genes, and genes associated with regulatory and exhaustion phenotype, and EMT, including *PLS3* and *TWIST,* the loss of *STAT4* expression and the co-expression of oncogenic microRNAs. The identification of gene subsets that are unique to each disease will likely improve diagnostic accuracy. Functional studies with these genes will yield further insight into the pathogenesis of Sézary syndrome.

In conclusion, the Special Issue “Leukocytes in Inflammation, Resolution of Inflammation, Autoimmune Diseases and Cancer” presents a selection of studies that elucidate the diverse roles of leukocytes in inflammation, underlying a range of pathologies. Although leukocytes have been and still are intensely studied, the knowledge of many aspects of their functions, in particular those orchestrating repairs and the resolution of inflammation, is only beginning to emerge. A deeper understanding of how this dichotomy is regulated and to what extent these features can be exploited to promote resolution and repair in patients represent quite exciting lines of ongoing and future investigations.
